# Palliative Care and End-of-Life Issues in Patients with Brain Cancer Admitted to ICU

**DOI:** 10.3390/medicina59020288

**Published:** 2023-02-01

**Authors:** Sara Frisella, Lapo Bonosi, Mariachiara Ippolito, Giuseppe Roberto Giammalva, Gianluca Ferini, Anna Viola, Valentina Anna Marchese, Giuseppe Emmanuele Umana, Domenico Gerardo Iacopino, Antonino Giarratano, Andrea Cortegiani, Rosario Maugeri

**Affiliations:** 1 Department of Surgical, Oncological and Oral Science (Di.Chir.On.S.), University of Palermo, 90100 Palermo, Italy; 2 Neurosurgery Unit, Department of Biomedicine, Neurosciences & Advanced Diagnostics (BiND), School of Medicine, University of Palermo, 90127 Palermo, Italy; 3 Department of Anesthesia, Intensive Care and Emergency, Policlinico Paolo Giaccone, University of Palermo, 90100 Palermo, Italy; 4 Department of Radiation Oncology, REM Radioterapia SRL, 95125 Catania, Italy; 5 Gamma Knife Center, Trauma Center, Department of Neurosurgery, Cannizzaro Hospital, 95100 Catania, Italy

**Keywords:** palliative care, supportive care, neuro intensive care unit, neuro-ICU, end-of-life therapies, brain tumors, neurosurgery

## Abstract

*Background and Objectives*: Palliative care is an interdisciplinary medical specialty focused on improving the quality of life of critically ill patients, including those with frailty, during their illness. *Materials and Methods*: We conducted an extensive literature review on Pubmed focusing on palliative care in neuro-oncology patients admitted to intensive care units (ICUs). *Results*: We identified 967 articles and, after excluding 952 articles in accordance with the PRISMA flow chart, we included a total of 15 articles in the final selection. The potential role of palliative care in neuro-oncology appears necessary to ensure comprehensive end-of-life patient care. However, this seems underestimated and poorly applied, especially in the context of intensive care units. Medical personnel also face ethical dilemmas, considering not only the pathology but also the socio-spiritual context of the patient. In addition, caregivers’ understanding of prognosis and realistic goals is critical for optimal end-of-life management. *Conclusions*: The provision of palliative care to neuro-oncological patients admitted to ICU is a complex challenge supported by fragmented evidence. Additional research on palliative care and communication about end-of-life care in the neuro-oncology and neuro-ICU setting is needed.

## 1. Introduction

During the last years, several progresses have been achieved in the treatment of malignant brain tumors, combining surgery, chemotherapy and radiotherapy (e.g., in the case of high-grade gliomas) [1]. However, the prognosis of patients affected by such tumors remains poor, with a mean overall survival of about one year from first diagnosis (i.e., 14–15 months). Additionally, comorbid and frail patients may experience an even worse course [2], with difficult hospital stay, sometimes requiring postsurgical intensive care unit (ICU) admission. Peculiar management is expected for patients with anticipated poor prognosis, as aggressive therapeutical approaches would not determine a full restoration of organ function, and life expectancy may not be affected by curative care [3]. In this light, ICU admission may be considered inappropriate. When occurring, neuro-palliative care consultation can also be offered in an ICU setting. The main aim is to discuss the goal of care and management in the ICU (e.g., management of postoperative brain edema, transient respiratory and/or cardiovascular support) with patients and caregivers. Although the benefit of such an approach is reasonable in terms of burden reduction, the potential role of palliative care in neuro-oncology seems underestimated and poorly applied, especially in the setting of ICUs [4]. Caregivers’ understanding of the prognosis and realistic goals is pivotal for subsequent decision making, and hospice services may become an option for complex symptoms persistent after ICU discharge.

The aim of this narrative review was to describe the extent of palliative care application and end-of-life issues in neuro-oncological patients with particularly malignant or end-stage brain tumors admitted to the ICU.

## 2. Materials and Methods

For this narrative review, evidence was retrieved by a systematic search of the literature. The protocol of this review was prospectively registered in Open Science Framework, and it is publicly available online at https://doi.org/10.17605/OSF.IO/AT7PW. On 6 June 2022, a systematic literature search was performed in the electronic database of Pubmed. The review was conducted according to the Preferred Reporting Items for Systematic reviews and Meta-analyses guidelines and recommendations (PRISMA) [5]. We performed a broad systematic literature search in Pubmed for all studies investigating the topic of palliative care in neuro-oncological patients in the intensive care unit (ICU) setting. We searched for all studies published in the last 10 years, up to the 6th of June 2022, using the following MeSH and free-text terms: “palliative care”, “palliative therapy”, “palliative therapies”, “end of life therapies”, “end of life care”, “supportive care”, “supportive therapy”, “supportive therapies”, “neuro oncology”, “neuro ICU”, “neuro intensive care unit”, “neuro tumors”, “neuro cancers”, “ brain tumors” and “neurosurgery”, combined using Boolean operators “AND” and “OR”. The search query in its full version is available in Supplementary Material S1. To avoid the potential omission of relevant studies, we also manually screened reference lists of articles included and previous systematic reviews and meta-analyses regarding similar topics.

### 2.1. Study Selection

The first screening was based on the analysis of titles and abstracts. The article’s full text was retrieved for further investigation if the title and abstract met the inclusion criteria. Two authors (L.B. and S.F.) independently assessed eligibility based on prespecified criteria, and discrepancies were solved by arbitration of a third author (G.R.G.), and the final list of included articles was finally validated against the exclusion criteria by a fourth author (M.I.). The data collection process was conducted without using any automated tool.

### 2.2. Eligibility Criteria

The articles were selected according to the following inclusion criteria:

- Full Article in English;
- Clinical studies (case series, observational cohort studies, retrospective or prospective studies);
- Patients age ≥18;
- Patients affected by primary or secondary brain tumors;
- Studies focusing on palliative/supportive care in ICU setting;
- Studies assessing patients, caregivers, or medical staffs’ perspective about end-of-life (EoL) period.
- Exclusion criteria:
- Articles not in English;
- Editorials, literature reviews, systematic reviews and meta-analyses;
- Patients age ≥18;
- Oncological patients without brain involvement;
- Studies considering neurosurgical palliative treatments in oncological patients;
- Studies not assessing palliative or supportive care;
- Studies evaluating palliative care in an “extra-ICU” setting.

### 2.3. Data Collection and Presentation

Data were collected using electronic standardized forms and categorized based on the topic. They were presented as plain text and tables.

## 3. Results

**Figure 1:**
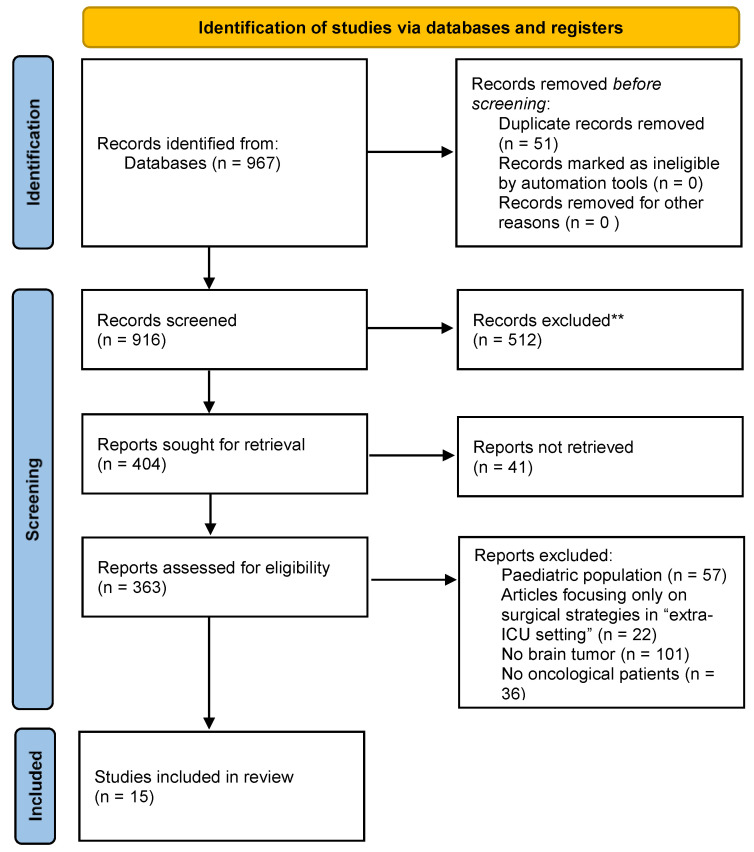
PRISMA flow chart.

From a total of 915 records retrieved, after duplicate exclusion and screening from abstracts, 363 relevant articles were screened from full text; of those, 15 articles [6,7,8,9,10,11,12,13,14,15,16,17,18,19,20] were adjudicated relevant for inclusion in this review and provided the basis for data and discussion presented on the topic (Table 1). Twelve studies were conducted in a single center, and only three were multicentric studies. Eleven studies were conducted on patients, and four studies included healthcare providers and/or caregivers. The full process of inclusion and exclusion is graphically presented at Figure 1.

## 4. Discussion

### 4.1. Setting Goals of Care in Neuro-Oncology

The management of the end-of-life phases of a neuro-oncological patient often requires a multidisciplinary approach, aimed at establishing the most appropriate treatment for the patient’s needs. This goal may be challenging, as patients may experience rapid worsening of cognitive functions and may not be able to express their will and intention early. Moreover, the caregivers’ emotional burden can contribute to make clinical decisions hard to take. Over the recent decades, indeed, treatments have become able to heavily affect short-term survival of patients, independently of the severity of the disease, thanks to advances in medical knowledge and research. However, several questions arose regarding whether such invasive treatments could be considered futile or appropriate [21]. The definition of futility has, indeed, a wide range of meanings, involving both quantity and quality of life, and being strongly related to both patients’ and physicians’ goals and expectations [21]. Sometimes, a lack of agreement or understanding between physicians and patients or caregivers may hamper trustful physician–patient relationships, with negative consequences for both psychological and legal aspects [21]. To this extent, competencies and knowledge in the setting of palliative care and communication become pivotal. Several factors can contribute to the choice of the most appropriate treatment, i.e., clinical background, patient primary illness [7], the availability of prognostic scores [13] and characteristics of the clinicians who care for the patient. Indeed, a recent study has assessed how frequently 36 attendings and 14 fellows perceived a patient was receiving futile treatments. The study pointed out that clinicians’ experience was an important factor, contributing to the predictiveness and appropriateness of such assessments. In detail, the attendings less frequently considered a treatment as futile (11% vs. 21%, *p* > 0.001) and, when futility was assessed by fellows, patients were less likely to die during hospital stay (51% vs. 68%, *p* = 0.003) than patients so assessed by attendings [17].

### 4.2. When and How to Palliative Care Consult

The available literature in the field of neuro-palliative care emphasizes the importance of standardized guidelines for end-of-life care in patients with extremely malignant primary brain tumors (such as high-grade glioblastoma), which in most of the cases have an invariably poor prognosis. Indeed, patients with primary brain tumors not only differ from patients with non-CNS tumors, but they also differ from patients with brain metastases in signs and symptoms and in psychological aspects. Patients with these primary brain tumors have poorer performance status, higher levels of nursing support and more family overburdening [22]. A study aimed to compare the aggressiveness of the end-of-life score of a cohort of non-CSN cancer group of patients with a cohort of patients with high-grade glioma (HGG) [7]. For the scoring, as previously validated, one point is given for each indicator of aggressiveness in the last 30 days of life: ≥2 emergency room visits, ≥2 hospital admissions, ≥14 days of hospitalization, ICU admissions, death in hospital and receipt of chemotherapy within the last 14 days of life. The score ranges from 0 to 6, with a higher score indicating aggressiveness of care. A “good” EoL care was defined as a score of 0. The study found that mean scores were higher in non-CNS cancer patients (1.34 ± 1.57) compared with the HGG patients having (0.65 ± 1.1) and not having received (0.69 ± 1.01) formal palliative care consultation (*p* = 0.007). This finding reflected a more aggressive care of patients with noncentral nervous system advanced cancer during end-of-life stages in comparison with patients with high-grade glioma [7]. Especially in neurocritically ill patients, who may survive with massive functional and cognitive impairment, an accurate discussion between clinicians, patient and caregivers must be led early on in order to define the quality of life that the patient would find acceptable [23]. From the time of admission, all the available prognostic information and treatment options should be discussed so that the patients’ wills and goals of care can be considered. It is, in this context, more difficult for clinicians to rely on specific “trigger events” (e.g., impairment of mental abilities) to estimate an average 30-day survival, as brain tumor patients often present cognitive impairment at diagnosis; thus, an early palliative care consulting (PCC) is essential in order to ensure them an end-of-life treatment appropriate to their wills [6].

PCC or advance care planning is a process to discuss and evaluate end-of-life goals according to patient wills [24]. An early PCC has been found to be associated with a lower ICU admission rate and with an increased family satisfaction regarding health care and patient death quality [24]. A study has also showed that a palliative care consult was performed in 28% of a cohort of critically ill patients with brain metastases. Interestingly, the overall mortality did not statistically differ between those who received a consult and those who did not (78.8% vs. 90.3%, *p* = 0.15), witnessing that no additional risk, but relevant benefits, may derive from palliative care in such patients (e.g., symptom management and assistance in complex medical decision making) [20].

Every patient may have personal priorities and beliefs and, according to them, goals of care can be established. For example, the use of opioids can be a great resource against pain, but it can also compromise daily mental faculties, such that some patient would prefer a lower level of pain management in order to keep their complete mental faculties unmodified [25].

### 4.3. Palliative Care Approaches

Once palliative care approach is adopted, during the whole ICU stay, it is mandatory to consider that neuro-ICU patients may be unable to communicate their distress; thus, clinicians must be particularly vigilant for signs of discomfort, e.g., dyspnea, sleep deprivation, incontinence, depression, delirium and anxiety. The assessment of these symptoms may be challenging, since tachycardia and/or tachypnea can be the only manifestation of distress. Treatment of such symptoms in these patients is similar to those of the general population [23]. According to a study conducted by Thier et al. about end-of-life needs of patients with high-grade glioma, the most used drug category was opioids, adopted in 95% of the cohort [22]. Indeed, medication availability for withdrawal of life support includes, bedside opioids, to manage agonal breathing patterns and other signs of dyspnea or distress. Anticholinergics can be used to reduce tracheobronchial secretions and gurgling, and anxiolytics should also be immediately available in order to treat anxiety or agitation [23].

Medications and interventions that do not offer symptoms relief, such as antibiotics, vasopressors and inotropes and antithrombotic agents, may be discontinued after collegial discussion among involved healthcare professionals and caregivers. The same discussion may regard intravenous hydration, artificial feeding and level of patient monitoring. Other medications, such as antiepileptics, must be continued since seizures are considered uncomfortable complications [23].

Two of the most common approaches to neurocritically ill patients are the “comfort measures only”, focusing on symptoms relief, and the “full life support”, which includes, for example, tracheostomy or feeding tube placement. Another common approach is the “no escalation of treatment”. When intermediate measures are proposed without adequately discussing the goals, confusion may be delivered to caregivers, and prolongation of the dying process may occur [23]. This may help interpreting conflicting data on palliative care consultations and their association with outcomes. As an example, it may be confusing to know that in a 10-year cohort of patients with primary brain tumors, patients receiving palliative care received less oncologic treatment (e.g., brain surgery, chemotherapy, or radiation) than those not receiving palliative care, but they were more likely to receive life-sustaining treatments (e.g., intubation, mechanical ventilation and nutritional support) [9]. Written protocols regarding the goals of care and the resuscitation dispositions [23] in the case of withdrawal of life support may be of practical help, together with a careful selection of timing of life support withdrawal according to caregivers’ readiness to accept end of life. Such issues may be more easily addressed using formal palliative care consultations. Indeed, a single-center retrospective study conducted in a neuro-ICU found that, in 88% of the cases of palliative care consultations, the need of assistance in defining a patient’s goals of care (88%) [19] was the main reason of the request. A similar result was reported by Tran et al., who reported “clarifying goals of care” as the main reason for palliative care consultation in 22 patients out of the 25 assessed, as well as other reasons, including assistance in “family support”, “decision making”, “discharge planning” and “communication” [10]. Many deaths among neurocritically ill patients occur chronologically after a decision to limit life-sustaining therapies [23]. According to this observation, withdrawal of life-sustaining therapies must be an option to consider and should not be interpreted as “causing death” but as “allowing the patient to die”.

Indeed, a single-center study reported that tumor progression was the most common cause of death in 77% of a cohort of patients with HGG. In this cohort, despite palliative care being implemented in 95% of patients, resuscitation was performed in 13% [14].

Unluckily, despite several studies showing that palliative care improves quality of life in patients with brain malignancy, PCCs are often underused in this category of patients [23]. Indeed, palliative care needs in patients admitted to neuro-ICUs are often unmet or met late. A retrospective study based on a chart review of patients who died in neuro-ICU found that 44% of expired patients never received palliative care referral, and that patients were, on average, referred only 1 day before death [8]. On the other hand, evidence exists for a high prevalence of palliative care needs in a neuro-ICU (62%), which may be detected if systematically screened [13].

### 4.4. Clinicians Point of View and Potential Issues

Thus, the issue of end of life, especially in the field of oncology, and particularly in neuro-oncology, still represents an ethical and professional dilemma, even for experienced healthcare providers facing this situation. However, a survey has shown that experienced neuro-oncology clinicians may have fewer moral objections to medical assistance to death [6], and that profession, gender, Catholicism, age of patients, clinical volume and whether the practice is allowed regionally did not predict moral objection [6].

The debate on the use of medical technology to prolong life regardless of the quality of outcomes often remains open and unresolved [21]. The acceptance of death by many healthcare professionals exemplifies an unacceptable bias, shaping itself as a personal defeat and leaving them at the mercy of a sense of inadequacy in the face of what is, in some ways, “beyond human”. In this context, scientific skill and knowledge collide with the ethics and culture peculiar to each individual and each country, raising issues of paramount importance in the comprehensive care of these kinds of patients. During end-of-life discussions, there are many complex concepts, and advanced care planning and consideration of the individual’s values appear crucial [26]. Healthcare professionals can play an essential role by providing detailed information about the advanced medical treatment used during end-of-life care. Physicians should perform their duties deontologically by providing patients with detailed information about a therapy’s benefits, limitations, and drawbacks [27].

Furthermore, physicians have the responsibility to preserve patient’s life, but this is not to be confused with unnecessary use of resources and inflicting more harm than good on the patient by continuing medically futile treatments [28,29]. For instance, Yan et al. [30] have proposed a new approach using the “Cardinal Issue Perspective” on decision making as a checklist for routinely performing shared decision making in end-of-life situations. This 10-point checklist is a comprehensive framework for managing decision processes and ensuring autonomy and quality. The first three cardinal issues (“Need”, “Mode” and “Investment”) are devoted to setting the stage for decision-making efforts, to discuss the urgency of the decision and the benefits and risks of watchful waiting vs. actively deciding and to assess the preferred level of involvement, discussing the content together to help patients and surrogate decisionmakers understand issues pertinent to decision making. The subsequent five cardinal issues (“Options”, “Possibilities”, “Judgment”, “Value” and “Tradeoffs”) reflect a shared decision-making approach. The remaining two (“Acceptability” and “Implementation”) concern the making of the final decision, dealing with potential objections from participants in the decision making. Decisions must be tailored to individual patients and have no generalized approach: one disease, one individual, one end-of-life care. Hence the need to consider not only the physical aspects but also the psychological, social and spiritual elements of each patient. Another critical aspect is sharing information on the patient’s condition; decisions about the amount and quality of information to be given to patients and caregivers are often complex and ongoing and should be recognized as a significant and challenging aspect of the clinical workload [16].

This is especially true for healthcare professionals working in neuro-ICUs, where a variable proportion of patients have life-limiting conditions; in this context, the need in such units for swift prognostication and relevant application of palliative care becomes crucial [31]. Recent models posit that end-of-life palliative care goes beyond simply symptom management to include psychosocial care, family support, spiritual assistance and even follow-up with bereavement services after death [32]. Bluck et al. [33] have identified three distinct perspectives on palliative care: holistic, comfort care and foundational perspectives. The first represents a concern for nuanced psychosocial and spiritual care; the second is focused on continued physical symptom and pain management up until death, while the last embodies theoretical understanding of palliative care. They underlined how a more significant endorsement of the holistic perspective predicted a greater acceptance of ethical care delivery and lowered futile care involvement, while a greater endorsement of the comfort care viewpoint favored early palliative care team involvement. Finally, strongly endorsing the foundational perspective was associated with a deeper understanding of palliative care principles. The extent to which practitioners supported each of the different perspectives was related to their end-of-life expertise. However, systemic barriers in neuro-ICUs daily work often impede the implementation of this expertise [34]. Among them, lack of care coordination, limited time or staffing, the length of procedures necessary for a referral, discordant views and interpretations among patients, caregivers and clinicians and narrow knowledge on palliative care principles are the most frequently encountered obstacles [15,34,35].

## 5. Conclusions

The provision of palliative care to neuro-oncological patients admitted to ICUs is a complex challenge supported by fragmented evidence. Additional research on palliative care and communication about end-of-life care in the neuro-oncology and neuro-ICU settings is needed, and practical guidance (i.e., protocols, formal palliative care services and consultations and guidelines) should be provided to clinicians. Consensus on the most adequate research study designs and core outcomes would also allow for improvements of knowledge in the field. Meanwhile, clinicians should consider early palliative care consultations, setting goals of care at ICU admission and the application of general palliative care principles in this specific patient population.

## Figures and Tables

**Table 1:** Characteristics of the included studies.

Author	Design	Population	Goals	Main Outcomes
Climans et al. [6]	Survey, single-center.	A total of 125 healthcare professionals involved in neuro-oncology.	To explore attitudes towards medical assistance in death (MAiD), interpretation of scenarios.	Questions on nine scenarios of brain cancer patients needing medical assistance in death.
Harrison et al. [7]	Observational retrospective study, single-center.	A total of 212 patients identified, including 80 HGG patients without PCC, 52 HGG patients with PCC and 80 non-CNS-primary cancer with PCC.	To evaluate end-of-life score, assessing aggressiveness of EoL care in the cohorts of included patients.	Aggressiveness of EoL care score; time to PC referral.
Morris et al. [8]	Observational retrospective study, single-center.	A total of 330 patients expired in neuro-ICU from both neurology and neurosurgery services.	To identify barriers, institutional or patient-related, to the provision of palliative care service.	Time to palliative care referral and arrival, time to hospice referral and arrival, time to withdrawal of curative care, time to expiration and thematic barriers to referral.
Kubendran et al. [9]	Observational retrospective multicentric study.	A total of 37,365 patients with primary brain malignancies in inpatient palliative care consult in a 10-year period.	To identify factors involved in inpatients’ palliative care in patients with PBMs.	Rates and risk factors for palliative care consultation over the study period.
Tran et al. [10]	Retrospective observational qualitative study, single-center.	A total of 25 neuro-ICU patients who underwent a PCC during their ICU stay.	To perform content analysis on the palliative care note and to identify main differences between patients with and those without PCC.	Reasons and issues addressed in PC consultations.
Mroz et al. [11]	Retrospective observational cohort study, single-center.	A total of 133 critically ill neurology and neurosurgery patients expired in a 2-year period in NICU.	To analyze the application of care and communication bundle in the ICU.	Barriers to care and communication bundles in the neuro-ICU.
Rosenberg et al. [12]	Retrospective observational cohort study, single-center.	A total of 90 patients with diagnosed HGG admitted to a neuro-ICU	To assess the incidence rate of an inpatient PCC, association between PCC and DNR status, length of stay, discharge dispositions, death within 30 days of admission, death location and 30-day readmission rate.	Incidence of inpatient palliative care consultation, code status amendment to do not resuscitate (DNR), discharge disposition, 30-day mortality and 30-day readmission rate, length of stay and place of death.
Creutzfeldt et al. [13]	Quality improvement project with a prospective parallel group cohort study, single-center.	A total of 130 patients from a neuro-ICU with implemented palliative care needs screening; a total of 132 patients from a neuro-ICU without implemented PC needs screening.	To identify palliative care needs for patients and their families and potential ways to meet those needs.	Prevalence and nature of palliative care needs and actions to address those needs through the use of four questions.
Barbaro et al. [14]	Retrospective observational cohort study, single-center.	A total of 132 adults with intracranial high-grade gliomas.	To understand patterns of care and circumstances surrounding end of life in patients with intracranial gliomas.	Causes and location of death, comfort measures and resuscitation effort.
Walsh et al. [15]	Prospective multicentric study.	A total of 17 patient, caregiver, and oncologist triads were analyzed.	To examine communication processes and goals among patients, caregivers and oncologists to elucidate drivers of prognostic understanding in the context of recurrent GBM.	Concordance between patient, caregiver and oncologist communication processes and goals.
Dumble et al. [16]	Prospective multicentric qualitative study.	A total of 10 prescribing clinicians (doctors and nurses).	To explore some of the many factors prescribing clinicians in the UK considered when deciding what information to give to EoL patients about medication choices and when.	Thematic factors.
Neville et al. [17]	Single-center retrospective comparative cohort study.	A total of 36 attendings and 14 fellows in intensive care units.	To explore prognostic ability among critical care fellows, comparing fellows’ and attendings’ assessments of futile critical care, and evaluate factors associated with assessments.	Frequency of futile treatment assessments and reasons
Habibi et al. [18]	Single-center retrospective observational cohort study.	A total of 145 patients diagnosed with brain metastases, including inpatients and admitted to ICU.	To study the effect of timing of palliative care (early vs. late) encounters on brain metastasis patients.	Palliative encounter, patient outcomes and healthcare utilization.
Pottash et al. [19]	Single-center retrospective observational cohort study.	A total of 55 patients admitted to the neurological ICU receiving a palliative care consultation.	To investigate the characteristics and impact of palliative care consultation for patients under the management of neurosurgical and critical care services.	Length of stay and mortality.
Kang et al. [20]	Single-center retrospective cohort analysis.	A total of 118 brain metastatic patients admitted to an intensive care unit.	To compare who received an inpatient palliative care consult with those who did not.	Mortality, time from intensive care unit admission to death, disposition and change in code status.

## Data Availability

Not applicable.

## Supplementary Materials

The following supporting information can be downloaded at: https://www.mdpi.com/article/10.3390/medicina59020288/s1, Supplementary Material S1: Search strategy.
