# Supplementary material for: Palliative Care and End-of-Life Issues in Patients with Brain Cancer Admitted to ICU
Source: Medicina (Kaunas). 2023 Feb 1;59(2):288. doi: 10.3390/medicina59020288 (PMC9962237; doi:10.3390/medicina59020288)
Supplement: Supplementary file 1 [file medicina-59-00288-s001.zip › medicina-2145940-supplementary.pdf]

Supplementary Material S1

Search: (("palliative care") OR ("palliative therapy") OR ("palliative therapies") OR("end of life care") OR ("end of life therapies") OR ("supportive care") OR("supportive therapy") OR ("supportive therapies")) AND (("neuro oncology") OR("neuro ICU") OR ("neuro intensive care unit") OR ("neuro tumors") OR ("neurocancer") OR ("neuro oncology") OR ("neurosurgery") OR ("brain tumors")) AND(y_10[Filter]) AND (y_10[Filter])
